# Prevalence of Budd-Chiari Syndrome during Pregnancy or Puerperium: A Systematic Review and Meta-Analysis

**DOI:** 10.1155/2015/839875

**Published:** 2015-09-20

**Authors:** Weirong Ren, Xiang Li, Jia Jia, Yan Xia, Fengrong Hu, Zhengyu Xu

**Affiliations:** ^1^Department of Digestive Diseases, Sanmenxia Central Hospital, Henan University of Science and Technology, Xiaoshan Road, Sanmenxia 472000, China; ^2^Xijing Hospital of Digestive Diseases, Xijing Hospital, Fourth Military Medical University, Xi'an 710032, China; ^3^Department of Emergency, Shanxi Provincial People's Hospital, Xi'an 710032, China; ^4^Assisted Reproductive Center, Shanxi Maternal and Child Care Service Hospital, Xi'an 710032, China; ^5^Department of Gastroenterology, No. 2 Hospital of Xi'an, Xi'an 710032, China

## Abstract

Women during pregnancy or puerperium are likely to develop Budd-Chiari syndrome (BCS). However, the reported prevalence of pregnancy-related BCS varied considerably among studies. Our study aims to systematically review this issue. Overall, 817 papers were initially identified via the PubMed, EMBASE, China National Knowledge Infrastructure, and Chinese Scientific and Technological Journal databases. Twenty of them were eligible. The prevalence of pregnancy-related BCS varied from 0% to 21.5%. The pooled prevalence was 6.8% (95% CI: 3.9–10.5%) in all BCS patients, 6.3% (95% CI: 3.8–9.4%) in primary BCS patients, and 13.1% (95% CI: 7.1–20.7%) in female BCS patients. Among them, one study was carried out in Africa with a prevalence of 10.6%; 14 studies in Asian countries with a pooled prevalence of 7.1% (95% CI: 3.1–12.6%); and 5 studies in European countries with a pooled prevalence of 5.0% (95% CI: 3.1–7.3%). The pooled prevalence was 6.7% (95% CI: 2.6–12.3%) in studies published before 2005 and 7.3% (95% CI: 4.2–12.5%) in those published after 2005. In conclusion, pregnancy is a relatively common risk factor for BCS, but there is a huge variation in the prevalence among studies. Physicians should be aware of pregnancy-related BCS.

## 1. Introduction

Budd-Chiari syndrome is characterized by hepatic venous outflow obstruction [1]. The main mechanism of obstruction is thrombosis of the hepatic veins (HV) or the terminal portion of the inferior vena cava (IVC) [2]. Recent studies have identified that many thrombophilic factors are associated with the development of BCS [3]. Common thrombophilic factors include inherited and acquired thrombophilia, such as antithrombin deficiency, protein C deficiency, protein S deficiency [4], heterozygous Factor V Leiden, prothrombin G20210A mutation [5], homozygous MTHFR mutation and hyperhomocysteinemia [6], and myeloproliferative neoplasms [7].

Pregnancy is a hypercoagulable state. The maternal hypercoagulable state is a physiological preparation for delivery; however, this hypercoagulability is associated with an increased risk of venous thromboembolism (VTE) [8–10]. The incidence of VTE in pregnant women, as derived from retrospective cohort studies, is estimated to be 5–12 events per 10,000 pregnancies antenatally (from conception to delivery), 7 to 10 times higher than the incidence in age-matched controls [10]. Clinical evidence also confirms that a pregnant woman's risk of VTE further increases immediately after the birth of the baby [11–15]. In a large population-based case control study from the Netherlands, a sixty-fold increase in the risk of VTE was detected in the puerperium compared with nonpregnant controls [16].

To date, the increased incidence of BCS in pregnancy or puerperium women suggests the possibility of a close relationship between pregnancy and BCS [3, 17–35]. However, the reported prevalence of pregnancy-related BCS (i.e., BCS happening during pregnancy or puerperium) in the literature has varied considerably. The present systematic review and meta-analysis primarily aim to evaluate the prevalence of pregnancy-related BCS from different regions.

## 2. Methods

### 2.1. Search Strategy

We searched the PubMed, EMBase, China National Knowledge Infrastructure (CNKI), and Chinese Scientific and Technological Journal databases (VIP) databases for studies that reported the prevalence of BCS during pregnancy or puerperium. The last search was performed on April 28, 2014. The search items were as follows: (“Budd-Chiari syndrome” or “hepatic venous outflow obstruction” or “hepatic venous outflow occlusion” or “membranous occlusion of inferior vena cava” or “membranous obstruction of inferior vena cava” or “hepatic vein thrombosis” or “hepatic vein occlusion” or “hepatic vein obstruction”) and (“pregnancy” or “postpartum” or “puerperium” or “peripartum” or “perinatal” or “obstetric*∗*”). The reference lists of the articles included were further hand-searched to identify any additional relevant studies.

### 2.2. Selection Criteria

The following inclusion criteria were used to identify target studies. (i) All observational studies, including cohort and case-control studies, were included; case reports, reviews, comments, essays, and animal studies were excluded. (ii) All studies reported the proportion of pregnancy-related BCS. (iii) The number of participants in all included studies was greater than 10. Studies with fewer than 10 patients were excluded because a small sample size could maximize the impact of systematic error, yielding less than authentic results. (iv) There were no publication date or publication status restrictions. (v) There were no language restrictions.

### 2.3. Data Extraction

All relevant studies were abstracted. A data extraction sheet was developed that included the authors, publication year, country, and affiliation where the study was carried out, study design, eligibility criteria, period of enrollment, number of patients with BCS, site of obstruction (IVC, HV, or combined obstruction), number of patients with pregnancy-related BCS, proportion of pregnancy-related BCS in total and female patients, and risk factors for the development of pregnancy-related BCS.

### 2.4. Quality Assessment

The studies were considered to be of higher quality if they fulfilled all of the following predetermined criteria: (1) patients were consecutively admitted; (2) interval of enrolment and eligibility criteria were clearly recorded; (3) patients were diagnosed with primary BCS; and (4) the sites of BCS obstruction were clearly reported.

### 2.5. Statistical Analysis

The prevalence of pregnancy-related BCS in each study was combined to yield a pooled prevalence with a 95% confidence interval (CI) for all studies. Data were pooled using a random-effects model to generate a more conservative estimate of the prevalence. Heterogeneity between studies was assessed using the *I* ^2^ statistic and the *χ* ^2^-test (*I* ^2^ > 50% or *P* < 0.10 was considered to indicate statistically significant heterogeneity). Publication bias was measured using Egger's test (*P* < 0.05 represents statistically significant publication bias). To explore the cause of heterogeneity among studies, subgroup analyses were performed according to the continents, publication years, and obstruction sites. Statistical analyses were carried out using the StatsDirect statistical software version 2.8.0 (StatsDirect Ltd, Sale, Cheshire, UK).

## 3. Results

### 3.1. Description of the Included Studies

**Figure 1:**
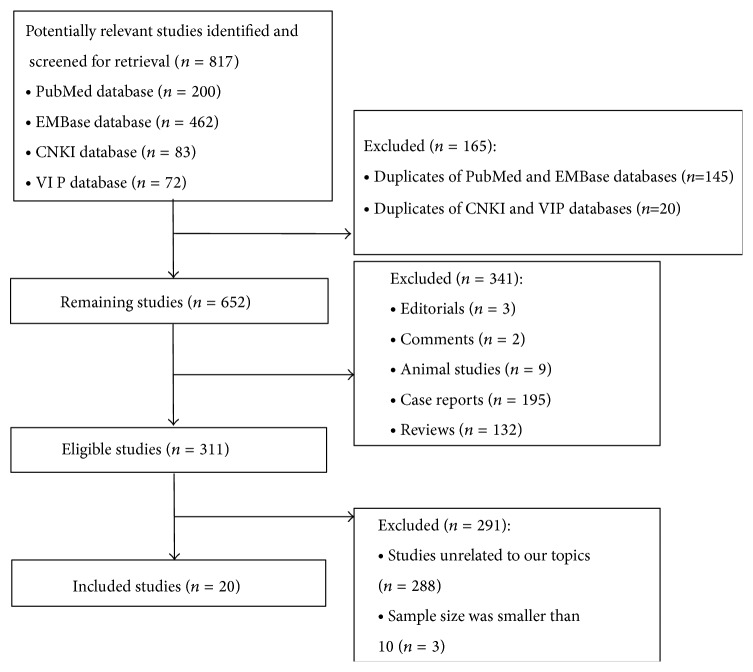
Flowchart for the literature search. Abbreviations: CNKI, China National Knowledge Infrastructure; VIP, Chinese Scientific and Technological Journal.

The search strategy identified 817 potentially relevant studies. Finally, 20 were eligible for the meta-analysis after the title/abstract screening and full-text screening (Figure 1) [3, 17–35]. All of these studies were retrospective cohort studies. Nineteen of the included studies were published in full-text form, and one was an abstract from an international meeting [17]. The detailed characteristics of these included studies are outlined in Table 1. Information regarding the eligibility criteria is shown in Supplementary Table 1 in Supplementary Material available online at http://dx.doi.org/10.1155/2014/839875.

### 3.2. Study Quality

Three (15.0%) studies were considered to be of high-quality [3, 23, 31] and 4 (20.0%) were of poor-quality (Supplementary Table 2) [24–26, 35]. Patients were consecutively admitted in 6 studies [17–20, 23, 31]. The intervals of enrolment and eligibility criteria were given by all included studies. Patients were diagnosed with primary BCS in 7 studies [18, 19, 22, 23, 27, 30, 31]. The sites of BCS obstruction were clearly reported in 12 studies [3, 19, 21–23, 27–29, 31–34].

### 3.3. Prevalence of Pregnancy-Related BCS

**Figure 2:**
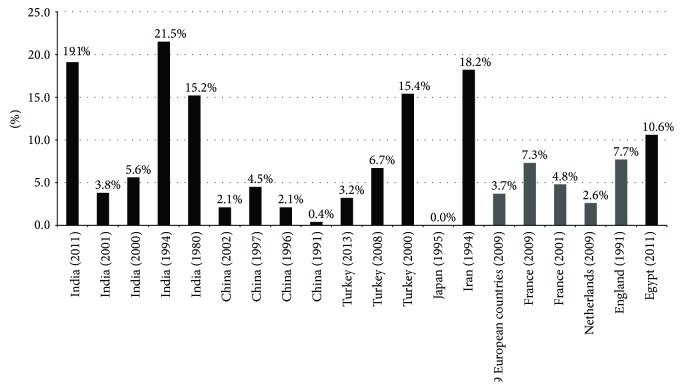
Prevalence of pregnancy-related BCS in different countries.

**Figure 3:**
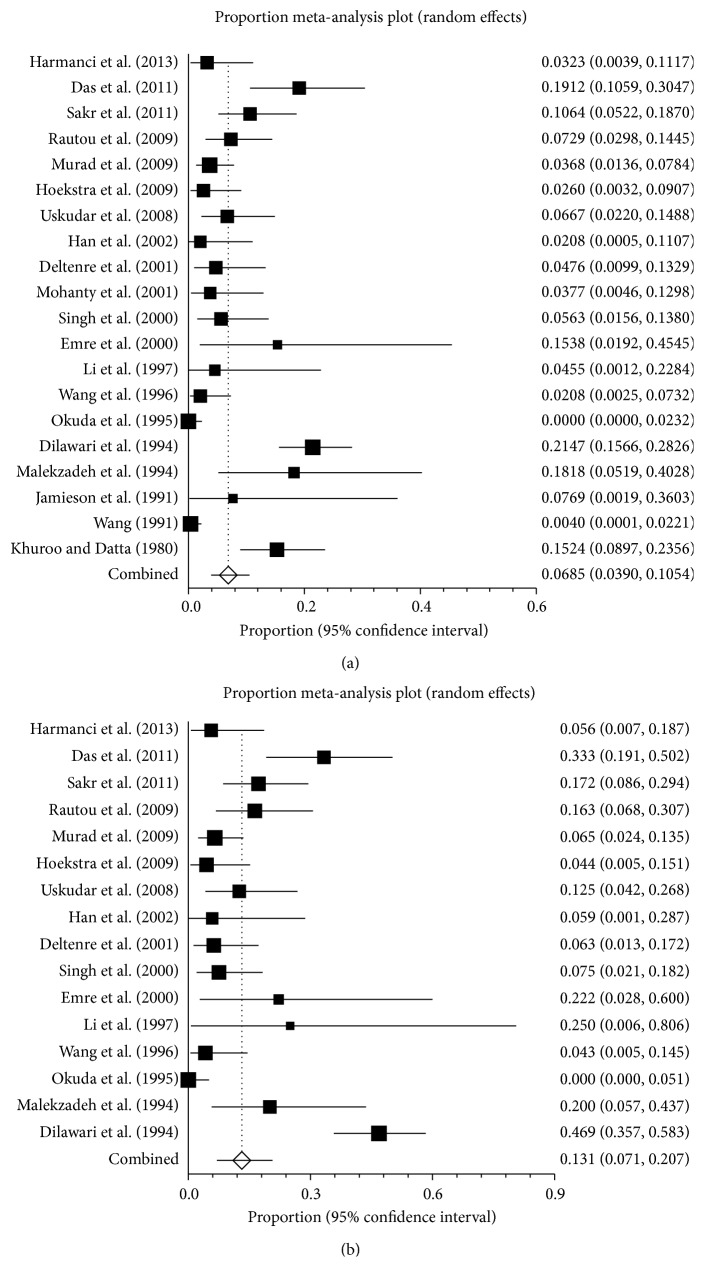
Forest plots of prevalence of pregnancy-related BCS in all included studies (a), female patients (b).

*Total Patients.* The prevalence of pregnancy-related BCS varied from 0 to 21.5% in 20 studies (Figure 2). The pooled prevalence was 6.8% (95% CI: 3.9–10.5%), with a statistically significant heterogeneity among studies (*I* ^2^ = 86.1%, 95% CI: 80.1–89.7%, *P* < 0.0001) (Figure 3(a)). The publication bias was statistically significant (Egger: bias = 2.46, 95% CI: 1.48–3.43, *P* < 0.0001).

*Female Patients.* Sixteen studies, each of which included the number of female patients with BCS, reported the prevalence of pregnancy-related BCS in female patients, ranging from 0 to 46.9% [3, 17–23, 26, 27, 29–34]. The pooled prevalence was 13.1% (95% CI: 7.1–20.7%), with a statistically significant heterogeneity among studies (*I* ^2^ = 86.0%, 95% CI: 78.8–89.9%, *P* < 0.0001) (Figure 3(b)). Publication bias was statistically significant (Egger: bias = 2.99, 95% CI: 1.30–4.67, *P* = 0.0019).

*Primary BCS Patients.* Only patients with primary BCS were included in 7 studies [3, 18, 22, 23, 27, 30, 31]. A pooled prevalence was 6.3% (95% CI: 3.8–9.4%), with no statistically significant heterogeneity among studies (*I* ^2^ = 46.5%, 95% CI: 0–75.7%, *P* = 0.0818) (Supplementary Figure 1). Publication bias was statistically significant (Egger: bias = 2.20, 95% CI: 0.14–4.27, *P* = 0.0407).

### 3.4. Subgroup Analysis according to Continent

*Africa.* One study was conducted in Egypt [31]. This study was carried out between 2009 and 2011 and reported a prevalence of 10.6%.

**Figure 4:**
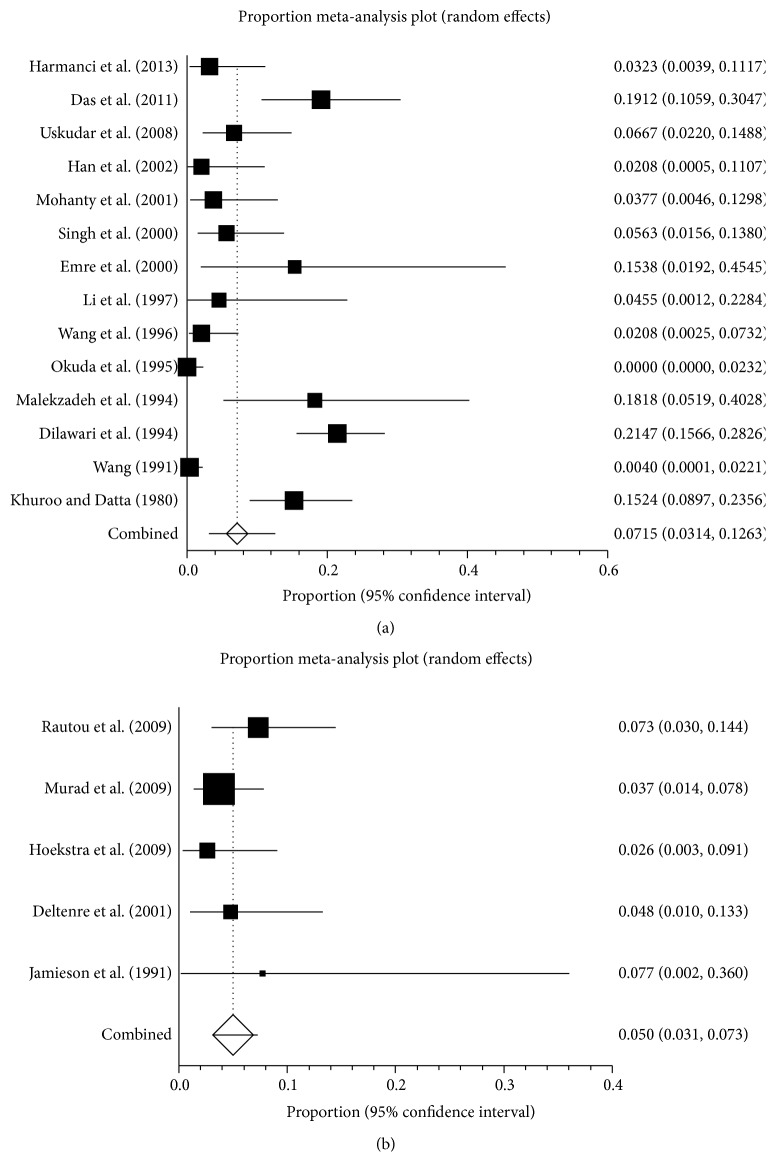
Forest plots of prevalence of pregnancy-related BCS in Asian (a) and European studies (b).

*Asia.* Fourteen studies were carried out in Asian countries, including India (*n* = 5) [17, 19, 25, 28, 32], China (*n* = 4) [21, 26, 34, 35], Turkey (*n* = 3) [20, 22, 33], Japan (*n* = 1) [29], and Iran (*n* = 1) [27]. These studies were carried out between 1963 and 2011 and reported a prevalence ranging from 0 to 21.5%. The pooled prevalence was 7.1% (95% CI: 3.1–12.6%), with a statistically significant heterogeneity among studies (*I* ^2^ = 90.0%, 95% CI: 85.4–92.6%, *P* < 0.0001) (Figure 4(a)). Publication bias was statistically significant (Egger: bias = 2.59, 95% CI: 1.24–3.95, *P* = 0.0013).

**Figure 5:**
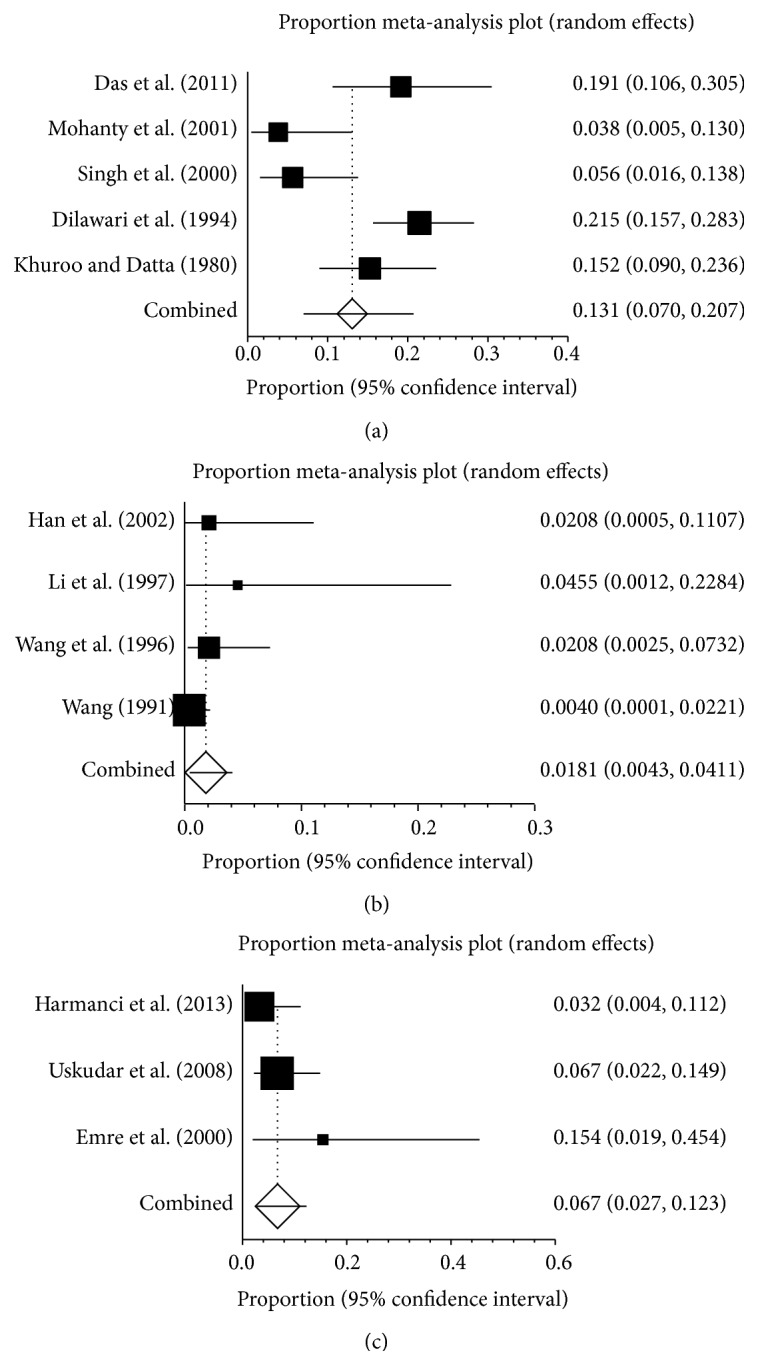
Forest plots of prevalence of pregnancy-related BCS in Indian (a), Chinese (b), and Turkish studies (c).

Five Indian studies were carried out between 1967 and 2000 and reported a prevalence ranging from 3.8 to 21.5% [17, 19, 25, 28, 32]. The pooled prevalence was 13.1% (95% CI: 7.0–20.7%), with a statistically significant heterogeneity among studies (*I* ^2^ = 79.5%, 95% CI: 34.3–89.6%, *P* = 0.0006) (Figure 5(a)). There was no proof of publication bias (Egger: bias = 5.48, 95% CI: −16.27–27.23, *P* = 0.4812).

Four Chinese studies were carried out between 1982 and 2002 and reported a prevalence ranging from 0.4 to 4.5% [21, 26, 34, 35]. The pooled prevalence was 1.8% (95% CI: 0.4–4.1%), without any statistically significant heterogeneity among studies (*I* ^2^ = 37%, 95% CI: 0–78.4%, *P* = 0.1898) (Figure 5(b)). The publication bias was statistically significant (Egger: bias = 0.90, 95% CI: 0.11–1.70, *P* = 0.0393).

Three Turkish studies were carried out between 1989 and 2011 and reported a prevalence ranging from 3.2 to 15.4% [20, 22, 33]. The pooled prevalence was 6.7% (95% CI: 2.7–12.3%), without any statistically significant heterogeneity among studies (*I* ^2^ = 24.9%, 95% CI: 0–79%, *P* = 0.0006) (Figure 5(c)). There was no proof of publication bias (Egger: bias = 5.48, 95% CI: −16.27–27.23, *P* = 0.264).

One Japanese study was carried out between 1975 and 1989 and reported a prevalence of 0% [29].

One Iranian study was carried out between 1984 and 1994 and reported a prevalence of 18.2% [27].

*Europe.* Five studies were carried out in European countries, including France (*n* = 2) [18, 30], England (*n* = 1) [24], the Netherlands (*n* = 1) [23], and multiple European countries (*n* = 1) [3]. These studies were carried out between 1976 and 2005 and reported a prevalence ranging from 2.6 to 7.7%. The pooled prevalence was 5.0% (95% CI: 3.1–7.3%), without any statistically significant heterogeneity among studies (*I* ^2^ = 0%, 95% CI: 0–64.1%, *P* = 0.5684) (Figure 4(b)). There was no proof of publication bias (Egger: bias = 0.85, 95% CI: −1.46–3.15, *P* = 0.3274).

Two French studies were carried out between 1994 and 2005 and reported a prevalence of 4.8% and 7.3% [18, 30]. One English study was carried out between 1976 and 1990 and reported a prevalence of 7.7% [24]. One Netherlands study was carried out between 2003 and 2005 and reported a prevalence of 2.6% [23]. One study was conducted by the Europe Network for Vascular Disorders of the Liver (EN-Vie) project between 2003 and 2005, which was composed of 9 European countries (France, Spain, Italy, Great Britain, Germany, Belgium, Netherlands, Portugal, and Switzerland); the reported prevalence was 3.7% [3].

### 3.5. Subgroup Analysis according to Publication Year

**Figure 6:**
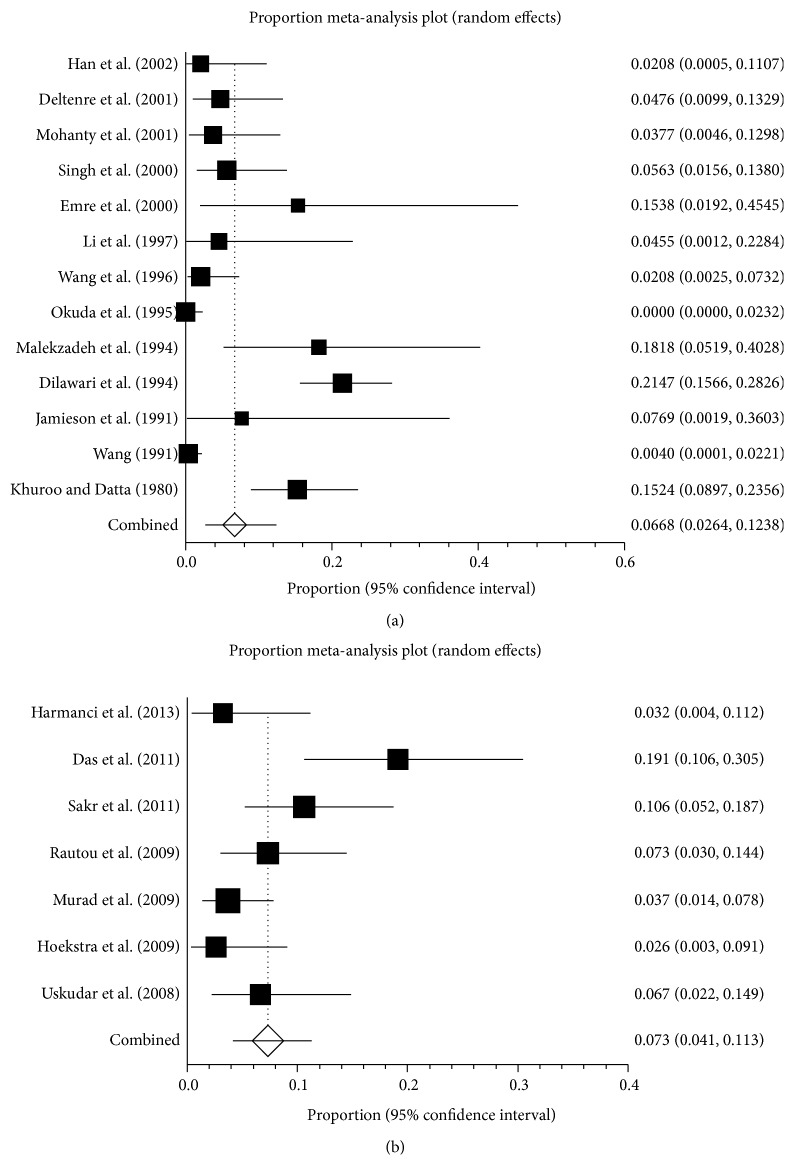
Forest plots of prevalence of pregnancy-related BCS according to the publication year ((a): before 2005, (b): after 2005).

Thirteen studies were published before 2005 [18–21, 24–29, 32, 34, 35]. The sample sizes varied from 13 to 250, and the prevalence was reported to range from 0 to 21.5%. The pooled prevalence was 6.7% (95% CI: 2.6–12.3%), with a statistically significant heterogeneity among studies (*I* ^2^ = 89.6%, 95% CI: 84.5–92.5%, *P* < 0.0001) (Figure 6(a)). The publication bias was statistically significant (Egger: bias = 2.26, 95% CI: 0.82–3.70, *P* = 0.0053).

Seven studies were published after 2005 [3, 17, 22, 23, 30, 31, 33]. The sample sizes varied from 62 to 163, and the prevalence was reported to range from 2.6 to 19.1%. The pooled prevalence was 7.3% (95% CI: 4.2–12.5%), with a statistically significant heterogeneity among studies (*I* ^2^ = 67.8%, 95% CI: 0.1–83.6%, *P* = 0.0049) (Figure 6(b)). Publication bias was statistically significant (Egger: bias = 3.74, 95% CI: 1.03–6.45, *P* = 0.0164).

### 3.6. Subgroup Analysis according to the Obstruction Sites

In twelve studies, data on the obstruction sites of BCS could be extracted. In six of them, the percentage of patients with IVC obstruction alone and IVC-HV combined obstruction was >70% [19, 21, 27, 29, 32, 34]. The pooled prevalence was 6.4% (95% CI: 0.8–16.8%), with a statistically significant heterogeneity among studies (*I* ^2^ = 93.3%, 95% CI: 88.8–95.5%, *P* < 0.0001) (Supplementary Figure 2(a)). There was no proof of publication bias (Egger: bias = 3.13, 95% CI: −0.78–7.04, *P* = 0.0902).

In only one study, the percentage of patients with HV obstruction alone was >70%, with a prevalence of 10.6% [31].

In the remaining 5 studies, the percentages of patients with IVC obstruction alone and IVC-HV combined obstruction were nearly equivalent to those of patients with HV obstruction alone [3, 22, 23, 28, 33]. The pooled prevalence was 4.4% (95% CI: 2.7–6.5%), without any statistically significant heterogeneity among studies (*I* ^2^ = 0%, 95% CI: 0–64.1%, *P* = 0.815) (Supplementary Figure 2(b)). There was no proof of publication bias (Egger: bias = 0.77, 95% CI: −2.24–3.77, *P* = 0.4763).

## 4. Discussion

Pregnancy is a hypercoagulable state with increased serum clotting factors, predominantly originating from the liver [36–38]. Thus, the concentrations of HVs and IVC might be higher in pregnant or puerperal women, resulting in their increased chance of developing thrombosis within the hepatic venous outflow. To our knowledge, this study is the first systematic review and meta-analysis to evaluate the prevalence of BCS during pregnancy or puerperium. An important finding of our study is that the pooled prevalence of pregnancy-related BCS is 6.8% in all BCS patients and 13.1% in female BCS patients, suggesting that pregnancy might be a relatively common etiology of BCS. It appears that the prevalence of pregnancy-related BCS (6.8%) is similar to that of other common risk factors, such as inherited antithrombin (2.3%), protein C (3.8%), and protein S (3.0%) deficiencies [4], prothrombin G20210A mutation (3.0%) [5], and factor V Leiden mutation (12.0%) in BCS [3]. Therefore, pregnancy must not be neglected as the etiology of BCS is being assessed.

The heterogeneity remained statistically significant among studies. To explore the causes of heterogeneity, subgroup analyses were conducted according to the following factors: continents, publication years, and sites of obstruction. This behavior of subgroup stratification could be explained by the following reasons. (i) The distribution of the BCS etiologies might be different due to geographic regions. Indeed, an observational study has found that common thrombophilia is less frequently found in Chinese BCS patients than in western BCS patients [39]. This discrepancy may account for the lower prevalence of pregnancy-related BCS in Chinese studies than in European studies (1.8% versus 5.0%). (ii) The courses of puerperium may be variable among countries. In some Asian countries, prolonged rest postpartum is followed by late and slow mobilization after childbirth. According the report by Dilawari et al., in rural India, puerperal women often experience up to 30 or 40 days of confinement and fluid restriction [19]. Thus, the combination of increased clotting factors, lack of activity, and dehydration may constitute a condition conducive to venous thrombotic complications, potentially leading to a higher prevalence of pregnancy-related BCS in Indian studies (13.1%). (iii) Our understanding of the relationship between pregnancy and BCS has improved over time. Thus, differences in the prevalence of pregnancy-related BCS might be observed based on publication years. As our study has shown, the pooled prevalence of pregnancy-related BCS in studies published before 2005 year was 6.7% (95% CI: 2.6–12.3%), compared with 7.3% (95% CI: 4.2–12.5%) in those published after 2005. (iv) IVC obstruction usually runs a chronic course; by contrast, HV thrombosis is often acute. Studies by Dilawari et al. and Rautou et al. have suggested that pregnancy-related BCS is usually an acute disorder with hepatic vein obstruction [19, 30]. Thus, performing a subgroup analysis stratified according to the sites of obstruction seemed to be worthwhile. Indeed, our study found a relatively higher prevalence of pregnancy-related BCS in studies with a larger proportion of patients with HV obstruction alone than in those with a larger proportion of patients with IVC obstruction. Still, it should be noted that this difference was slight, as the 95% CI values overlapped between the subgroups.

Several strengths can be considered from this study. First, the prevalence of pregnancy-related BCS was systematically reviewed in this paper for the first time. The increasing available data lends support that improved outcome of BCS may result from deep understanding and advanced management of underlying etiology [1, 2]. Based on this consideration, we conducted the study to further replenish the series of studies regarding the etiology of BCS [4–6, 39–43]. Second, an exhaustive search strategy was used to maximize the likelihood of identifying all relevant literature. In our previous studies, we had systematically searched the literature regarding BCS in PubMed, EMBASE, and Cochrane library databases [44, 45]. However, the data in Chinese BCS patients was extremely lacking in English-language literatures. To update and enrich the data, we further searched PubMed, EMBASE, and two comprehensive Chinese-language databases (i.e., VIP and CNKI databases) to systematically review the prevalence of pregnancy-related BCS. Third, a random-effects model was used to pool the data to provide a more conservative estimate of the prevalence.

Our study had several limitations. First, despite the extensive search strategy, the number of studies included in our meta-analysis was insufficient, thereby precluding the achievement of adequate statistical power. Second, the heterogeneity among the included studies was significant. Although we attempted to explore the causes of heterogeneity by performing subgroup analyses, the heterogeneity among the studies persisted. Therefore, the present findings should be explained with caution. Third, there were no sufficient data provided to perform a meta-analysis of the potential risk factors for pregnancy-related BCS. Of the 20 included studies, 19 had no information on this subject. Only Rautou et al. described any risk factor for pregnancy-related BCS other than underlying thrombophilia [30]. Future studies should explore this aspect more thoroughly.

In conclusion, this systematic review and meta-analysis demonstrated a global prevalence of pregnancy-related BCS of 6.8%. Accordingly, we recommended that the physicians should be highly vigilant against pregnancy-related BCS. Still, it should be noted that this prevalence varied considerably among studies. Despite the subgroup analyses performed according to the geographic regions, publication years, and obstruction sites of BCS, the sources of heterogeneity remained unclear. Further research should explore the risk factors for pregnancy-related BCS and their potential mechanisms.

## Supplementary Material

Supplementary Figure 1: Forest plots of prevalence of pregnancy-related BCS in primary BCS patients.
Supplementary Figure 2: Forest plots of prevalence of pregnancy-related BCS according to the obstruction sites ((a): the percentage of patients with IVC obstruction alone and IVC-HV combined obstruction was >70%, (b): the percentage of patients with IVC obstruction alone and IVC-HV combined obstruction were nearly equivalent to those of patients with HV).
Supplementary Table 1: Quality assessment.
Supplementary Table 2: Affiliations and eligibility criteria in the included studies.

## Figures and Tables

**Table 1:** Overview of the included studies.

Authors (year)	Journal	Country	Enrollment period	Number of patients with BCS	Sex, M/F	Site of obstruction (BCS)			Number of patients with pregnancy-related BCS	Prevalence of pregnancy-related BCS	Time of BCS diagnosis
						HV	IVC	Combined			
Harmanci et al. (2013) [22]	Journal of Clinical Gastroenterology	Turkey	1989.1–2011.5	62	26/36	35	8	19	2	3.2% (2/62) 5.6% (2/36^†^)	During pregnancy

Das et al. (2011) [17]	Hepatology International	India	Last 6 years	68	29/39		NA		13	19.1% (13/68) 33.3% (13/39^†^)	During pregnancy in 5 patients; during puerperium in 8 patients

Sakr et al. (2011) [31]	World Journal of Gastroenterology	Egypt	2009.4–2011.2	94	36/58	70	3	21	10	10.6% (10/94) 17.2% (10/58^†^)	During pregnancy

Rautou et al. (2009) [30]	Gut	France	1995.1–2005.12	96	NA		NA		7	7.3% (7/96) 16.3% (7/43^*^)	During pregnancy or puerperium

Murad et al. (2009) [3]	Annals of Internal Medicine	9 European countries^&^	2003.10–2005.10	163	70/93	80	4	79	6	3.7% (6/163) 6.5% (6/93^†^)	Pregnancy within 3 months before diagnosis

Hoekstra et al. (2009) [23]	Journal of Hepatology	Netherlands	2003.10–2005.10	77	32/45	38	1	38	2	2.6% (2/77) 4.4% (2/45^†^)	During pregnancy

Uskudar et al. (2008) [33]	World Journal of Gastroenterology	Turkey	2002.2–2004.4	75	35/40	35	23	17	5	6.7% (5/75) 12.5% (5/40^†^)	During pregnancy

Han et al. (2002) [21]	Journal of Hebei Medical College for Continuing Education	China	1993.1–2002.3	48	31/17	2	33	13	1	2.1% (1/48) 5.9% (1/17^†^)	During pregnancy

Deltenre et al. (2001) [18]	Gut	France	1994–1999	63	15/48	NA	11	NA	3	4.8% (3/63) 6.3% (3/48^†^)	During pregnancy

Mohanty et al. (2001) [28]	Hepatology	India	1995–2000	53	NA	19	18	16	2	3.8% (2/53)	During pregnancy

Emre et al. (2000) [20]	The American Journal of Surgery	Turkey	1993–1999	13	4/9		NA		2	15.4% (2/13) 22.2% (2/9^†^)	During Puerperium

Singh et al. (2000) [32]	Journal of Gastroenterology and Hepatology	India	1992–1997	71	18/53	20	10	41	4	5.6% (4/71) 7.5% (4/53^†^)	During pregnancy

Li et al. (1997) [26]	Chinese Journal of Clinical Gastroenterology	China	Last 10 years	22	18/4		NA		1	4.5% (1/22) 25.0% (1/4^†^)	During puerperium

Wang et al. (1996) [34]	Henan Journal of Diagnosis and Therapy	China	1990.6–1993.8	96	49/47	5	91	NA	2	2.1% (2/96) 4.3% (2/47^†^)	During pregnancy

Okuda et al. (1995) [29]	Journal of Hepatology	Japan	1975.1–1989.12	157	87/70	9	20	126^§^	0	0% (0/157) 0% (0/70^†^)	During pregnancy

Dilawari et al. (1994) [19]	Medicine	India	1967–1991.12	177	96/81	28	24	67^▲^	38	21.5% (38/177) 46.9% (38/81^†^)	During pregnancy or early puerperium

Malekzadeh et al. (1994) [27]	Iranian Journal of Medical Sciences	Iran	1984.6–1994.12	22	2/20	6	6	4	4	18.2% (4/22) 20.0% (4/20^†^)	During pregnancy or puerperium

Jamieson et al. (1991) [24]	Annales de Chirurgie	England	1976.11–1990.5	26	NA		NA		1	7.7% (1/13^*※*^)	During pregnancy

Wang (1991) [35]	Chinese Journal of cardiovascular and pulmonary diseases	China	1982.12–1991.3	250	NA		NA		1	0.4% (1/250)	During pregnancy

Khuroo and Datta (1980) [25]	American Journal of Medicine	India	1963–1978	105	NA		NA		16	15.2% (16/105)	During pregnancy or early puerperium

Abbreviations: BCS, Budd-Chiari syndrome; NA, not available; ^†^number of female with BCS; ^*∗*^number of female with BCS aged 15–45 years at diagnosis; ^&^includes France, Spain, Italy, Great Britain, Germany, Belgium, the Netherlands, Portugal, and Switzerland; ^§^the type of BCS is unclassifiable in 2 patients; ^▲^119 patients underwent percutaneous transhepatic hepatovenography and IVC catheterization with contrast study to evaluate the location of HV outflow occlusion; and ^*※*^possible etiological factors were identified in 13 cases.

## Abbreviations

BCS: Budd-Chiari syndrome
VTE: Venous thromboembolism
HV: Hepatic vein
IVC: Inferior vena cava.
