# Supplementary material for: Prevalence of Budd-Chiari Syndrome during Pregnancy or Puerperium: A Systematic Review and Meta-Analysis
Source: Gastroenterol Res Pract. 2015 Sep 20;2015:839875. doi: 10.1155/2015/839875 (PMC4592727; doi:10.1155/2015/839875)
Supplement: Supplementary file 1 — Supplementary Figure 1: Forest plots of prevalence of pregnancy-related BCS in primary BCS patients. Supplementary Figure 2: Forest plots of prevalence of pregnancy-related BCS according to the obstruction sites ((a): the percentage of patients with IVC obstruction alone and IVC-HV combined obstruction was >70%, (b): the percentage of patients with IVC obstruction alone and IVC-HV combined obstruction were nearly equivalent to those of patients with HV). Supplementary Table 1: Quality assessment. Supplementary Table 2: Affiliations and eligibility criteria in the included studies. [file 839875.f1.pdf]

Supplementary Figure 1.

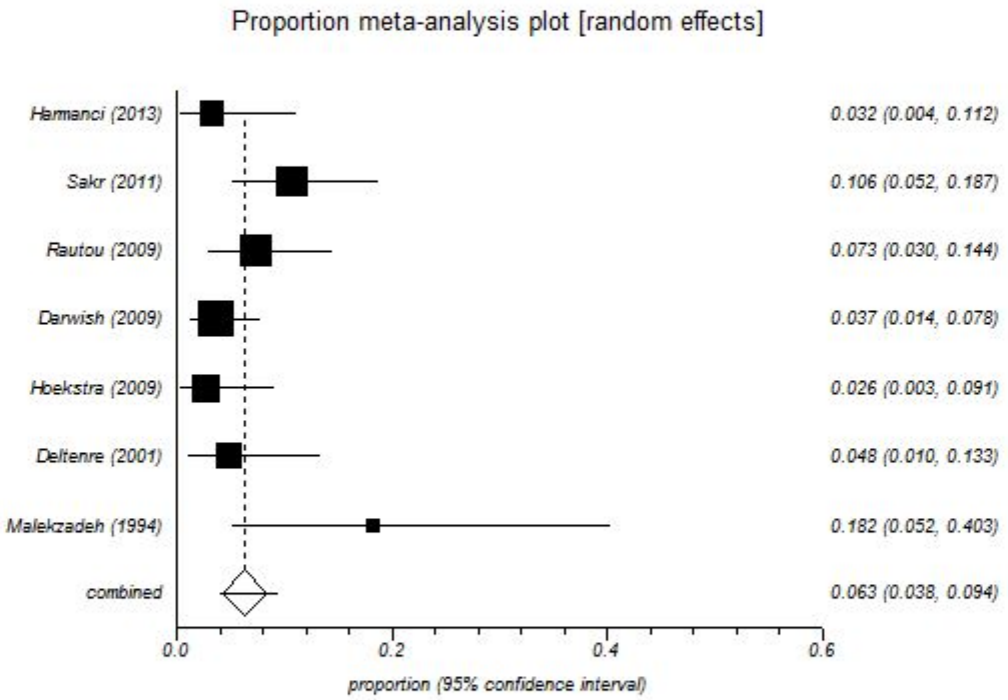

Supplementary Figure 2.

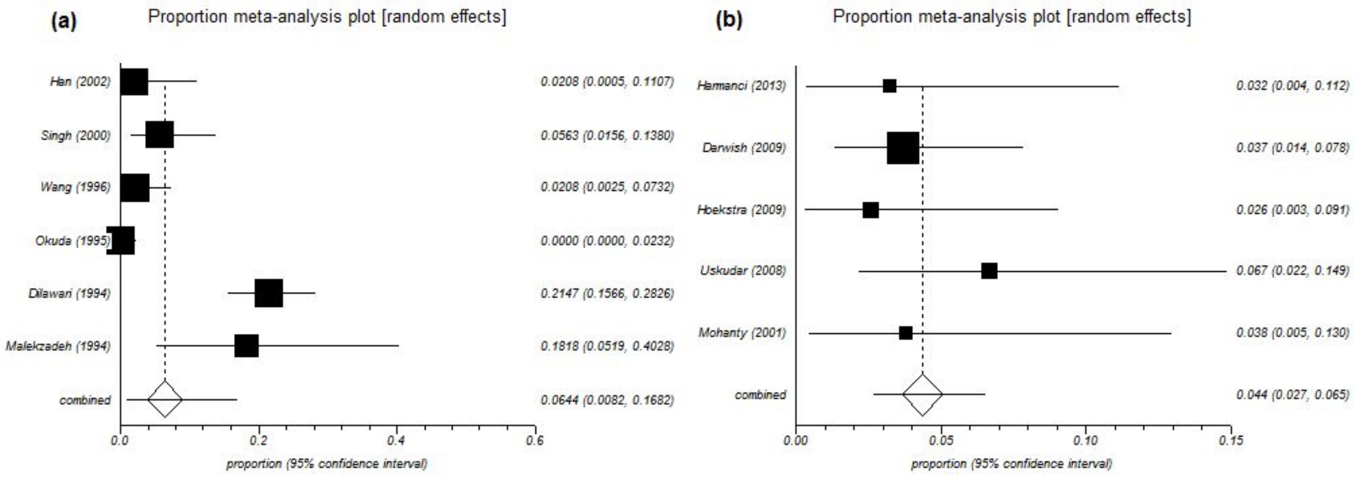

Supplementary Table 1. Quality assessment

Authors (year)	Item 1. Patients were consecutively admitted	Item 2. Interval of enrollment and eligibility criteria were clearly recorded	Item 3. Patients were diagnosed with primary BCS	Item 4. The sites of BCS obstruction were clearly reported	Total number of "YES"
Harmanci *et al.* (2013)	NA	Y	Y	Y	3
Das *et al.* (2011)	Y	Y	N	N	2
Sakr *et al.* (2011)	Y	Y	Y	Y	4
Darwish *et al.* (2009)	Y	Y	Y	Y	4
Hoekstra *et al.* (2009)	Y	Y	Y	Y	4
Rautou *et al.* (2009)	NA	Y	Y	N	2
Uskudar *et al.* (2008)	NA	Y	N	Y	2
Han *et al.* (2002)	NA	Y	N	Y	2
Deltenre *et al.* (2001)	Y	Y	Y	N	3
Mohanty *et al.* (2001)	NA	Y	N	Y	2
Singh *et al.* (2000)	NA	Y	N	Y	2
Emre *et al.* (2000)	Y	Y	N	N	2
Li *et al.* (1997)	NA	Y	N	N	1
Wang *et al.* (1996)	NA	Y	N	Y	2
Okuda *et al.* (1995)	NA	Y	N	Y	2
Dilawari *et al.* (1994)	NA	Y	N	Y	2
Malekzadeh *et al.* (1994)	NA	Y	Y	Y	3

Jamieson et al. (1991)	NA	Y	N	N	1
Wang *et al.* (1991)	NA	Y	N	N	1
Khuroo *et al.* (1980)	NA	Y	N	N	1

Abbreviations: Y, yes; N, no; NA, not available.

Supplementary table 2. Affiliations and eligibility criteria in the included studies

**Authors (year)**	**Design**	**Affiliations**	**Eligibility criteria**
Harmanci *et al.* (2013)	Original article	Hacettepe University School of Medicine Hospital	Inclusion criteria: BCS. Exclusion criteria: secondary BCS; congestive hepatic failure; sinusoidal obstruction syndrome; those lacking thrombophilia tests; patients refusing further diagnostic tests despite a diagnosis of BCS
Das *et al.* (2011)	Original article (Abstract)	Gastroenterology S.C.B. Medical College, Cuttack, Orissa, Cuttack India	Inclusion criteria: BCS
Sakr *et al.* (2011)	Original article	The Tropical Medicine Department of Ain Shams University Hospital, Cairo, Egypt	Inclusion criteria: primary BCS
Darwish *et al.* (2009)	Original article	9 European countries (France, Spain, Italy, Great Britain, Germany, Belgium, the Netherlands, Portugal, and Switzerland)	Inclusion criteria: BCS. Exclusion criteria: outflow obstruction occurred in the setting of heart failure, OLT, or hepatobiliary cancer.
Rautou *et al.* (2009)	Original article	Service d’He´matologie Biologique, Hoˆpital Beaujon, Clichy	Inclusion criteria: primary BCS
Hoekstra *et al.* (2009)	Original article	Department of Gastroenterology and Hepatology, University Medical Center Rotterdam	Inclusion criteria: BCS. Exclusion criteria: outflow obstruction occurred in the setting of heart failure, OLT, or hepatobiliary cancer.

Uskudar *et al.* (2008)	Original article	Turkiye Yuksek İhtisas Hospital	Inclusion criteria: BCS
Han *et al.* (2002)	Original article	No.2 Hospital of Baiding City, Hebei Province	Inclusion criteria: BCS
Deltenre *et al.* (2001)	Original article	Service d'He´matologie Biologique, Hoˆpital Beaujon, Clichy	Inclusion criteria: primary BCS (thrombosis of the hepatic veins or inferior vena cava)
Mohanty *et al.* (2001)	Original article	The Institute of Immunohaematology, Department of Gastroenterology KEM Hospital, Parel, Mumbai	Inclusion criteria: BCS
Emre *et al.* (2000)	Original article	Hepatopancreatobiliary Surgery Unit, and the Departments of Gastroenterology, and Radiology, Istanbul Faculty of Medicine, Istanbul University, Istanbul	Inclusion criteria: BCS treated by surgery
Singh *et al.* (2000)	Original article	Departments of Hepatology, Gastroenterology and Medicine, Postgraduate Institute of Medical Education and Research, Chandigarh	Inclusion criteria: BCS
Li *et al.* (1997)	Original article	The Forth people's Hospital of Luoyang, Henan province	Inclusion criteria: BCS
Wang *et al.* (1996)	Original article	Shandong Provincial Hospital	Inclusion criteria: BCS
Okuda *et al.* (1995)	Original article	50 institutes with more than 200 beds throughout Japan	Inclusion criteria: Japanese BCS cases. Tumor thrombus of IVC secondary to HCC was not excluded.
Dilawari *et al.* (1994)	Original article	The Department of Hepatic Diseases, Post-graduate Institute of Medical Education and Research, Chandigarh.	Inclusion criteria: BCS. Exclusion criteria: 1) patients in whom radiologic proof of diagnosis was not available during life; 2) hepatic veno-occlusive diseases; 3) neoplasia-related HV block demonstrated at autopsy; or 4) amebic liver abscess and venous lesions at

			autopsy.
Malekzadeh *et al.* (1994)	Original article	Dept. of Gastroent./Liver Disease, Shariati Hospital, Tehran Univ. of Medical Sciences, Tehran, Iran	Inclusion criteria: hepatic outflow obstruction. Exclusion criteria: tumors; congestive heart failure; or constrictive pericarditis.
Jamieson *et al.* (1991)	Original article	Department of Surgery, Addenbrooke's Hospital, Cambridge,	Inclusion criteria: BCS treated by liver transplantation
Wang et al. (1991)	Original article	Peking Union Medical College Hospital, Beijing Anzhen Hospital and Xinxiang Medical University	Inclusion criteria: BCS
Khuroo *et al.* (1980)	Original article	Nehru hospital, Post-graduate Institute of Medical Education and Research, Chandigarh.	Inclusion criteria: Patients with BCS following pregnancy
